# Evaluating body composition, the eating behavior scale, and the healthy lifestyle index in female Jordanian adults with metabolic syndrome: a cross-sectional study

**DOI:** 10.1186/s13098-025-01757-x

**Published:** 2025-08-07

**Authors:** Buthaina Alkhatib, Islam Al-Shami, Lana M. Agraib, Amjad Al Shdaifat

**Affiliations:** 1 Department of Clinical Nutrition and Dietetics, Faculty of Applied Medical Sciences, The Hashemite University, Zarqa, Jordan; 2 Department of Nutrition and Food Science, Faculty of Allied Medical Sciences, Al-Balqa’ Applied University, Al-Salt, Jordan; 3 Department of Internal and Family Medicine, Faculty of Medicine, The Hashemite University, Zarqa, Jordan

**Keywords:** Healthy lifestyle index, Eating behavior questionnaire, Metabolic syndrome, Body fat, Body muscle, BMI

## Abstract

**Background:**

Metabolic syndrome (MetS) is highly prevalent, and it is associated with unhealthy lifestyle risk factors that can be easily modified.

**Aims:**

To evaluate body composition, the adults’ eating behavior score (AEBQ), and the healthy lifestyle index (HLI) with metabolic syndrome (MetS) in female Jordanian adults.

**Methods:**

A cross-sectional study was conducted among 656 females in Jordan. Sociodemographic, anthropometric, blood pressure, and biochemical data were collected. The HLI and AEB questionnaire (AEBQ) was completed, and the waist-to-hip ratio (WHR) and body mass index (BMI) were calculated. MetS was determined based on NCEP-ATPII criteria.

**Results:**

Participants with MetS had significantly higher percentages of body fat (44.15 ± 6.37%) and body muscle mass (25.7 ± 4.56%). The mean HLI_BMI_ or HLI_WHR_ for participants with MetS (10.7 ± 1.51 and 11.6 ± 2.03, respectively) was significantly lower than for participants without MetS (11.5 ± 2.37, *p* = 0.003, and 12.4 ± 2.37, *p* = 0.004, respectively). Participants with five MetS components had significantly the highest % of body fat (46.07 ± 4.80, *p <* 0.001). The participants with zero components had significantly the highest HLI_BMI_ (12.97 ± 2.48, *p* = 0.003) and HLI_WHR_ (13.73 ± 2.39, *p* = 0.004). Regarding AEBQ, there was no significant variation between contributors with or without MetS.

**Conclusion:**

These findings suggest that as the number of MetS components increases, the % of body fat and body muscle mass also increases. This indicates a potential link between MetS and body composition. However, the scores for HLI_BMI_ and HLI_WHR_, indicators of a healthy lifestyle, decrease significantly as the number of MetS components increases. This highlights the importance of maintaining a healthy lifestyle to prevent or manage MetS.

## Plain language summary

Metabolic syndrome (MetS) is highly prevalent and is associated with unhealthy lifestyle risk factors that can be easily modified. We aimed to evaluate body composition, the adults’ eating behavior score (AEB), and the healthy lifestyle index (HLI) with (MetS) in female Jordanian adults through a cross-sectional study that included 656 females. Our findings suggest that as the number of MetS components increases, the percentage of body fat and body muscle mass also increases. This indicates a potential link between MetS and body composition. However, the scores for HLI_BMI_ and HLI_WHR_, indicators of a healthy lifestyle, decrease significantly as the number of MetS components increases. This highlights the importance of maintaining a healthy lifestyle to prevent or manage (MetS). (see Illustration graphical abstract).

**Graphical abstract:**

## Graphical abstract

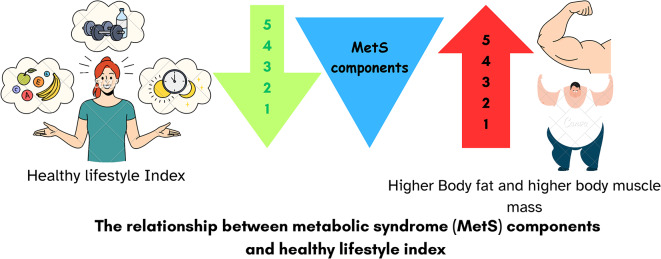

## Introduction

Metabolic syndrome (MetS) is a critical public health problem and has recently become of higher concern around the world. MetS stands for an assembly of metabolic abnormalities known as components that predispose a person to a greater risk of developing cardiovascular diseases (CVD) and type 2 diabetes (T2D) [1, 2]. The components of MetS are characterized by cardiometabolic risk, principally illustrated by central obesity, insulin resistance, dyslipidemia, hypertension, and hyperglycemia [3]. Additionally, MetS can be diagnosed if three or more of the five components — abdominal obesity, high blood pressure (BP), high fasting plasma glucose (FPG), hypertriglyceridemia, and low levels of high-density lipoprotein (HDL) cholesterol — are present simultaneously [4–6]. According to the provisional diagnostic conditions, the universal prevalence of MetS was reported to range from 12.5 to 31.4%, which demonstrated the extreme global prevalence of MetS and its components [7].

Several studies have documented the risk factors associated with the occurrence or development of MetS and its components; most of these factors are highly preventable and can be modified to decrease their prevalence or even prevent their occurrence. These modifiable lifestyle risk factors include increased weight and a higher body mass index (BMI) [8], higher fat mass index (FMI) levels, and body fat% % [9], decreased muscle mass [10], physical inactivity [11], smoking [12], unhealthy eating habits and dietary patterns [13]. Moreover, numerous investigations have confirmed that risk modification in routine life habits has been identified as a successful strategy for preventing MetS [13, 14]. Furthermore, combining these factors increased the risk of MetS occurrence [11].

Rather than concentrating on a single lifestyle risk factor, researchers have recently examined multiple risk factors in combination to assess the increased prevalence of metabolic syndrome (MetS). They generally analyzed ordinary physical activity, smoking cessation, healthy dietary patterns, and average weight as important issues of the healthy lifestyle score (HLS). A negative relationship was found between the HLS and the risk of MetS [15]. Likewise, adherence to the Healthy Eating Index (HEI), one of the components of the Healthy Lifestyle Score (HLS), has also been studied and found to be associated with a decreased occurrence of Metabolic Syndrome (MetS) [15]. On the other hand, adult eating behaviors questionnaires (AEBQ) were also used to assess the relationship between MetS and combined behaviors. Only slight variations in food consumption were reported between individuals with and without MetS, even though those with MetS scored lower on all evaluated eating behaviors. It was suggested that unhealthy eating habits led to poor diet quality rather than quantity [16].

In Jordan, MetS is highly prevalent; Ajlouni et al. found that in comparison to the 2009 survey, which found that the age-standardized prevalence rate of MetS was 34.6% in men and 39.8% in women, the 2017 rate was much higher (45.7% in men and 44.5% in women). Age, region, marital status, occupation, and gender were strongly correlated with MetS [17]. However, no studies have analyzed the effect of a mixture of lifestyle risk factors among the Jordanian population. The relationship between eating behaviors, lifestyle factors, and their consequences on MetS and its components remains unclear. This study aimed to investigate the association between HLI, AEBQ, and MetS and evaluate the relationship between body composition and MetS in adult Jordanian women.

## Materials and methodology

### Study design

A cross-sectional study was conducted in the Hashemite Kingdom of Jordan from January to March 2024. We invited a randomly selected sample from the population to participate in this study. The target sample was selected from the outpatient departments in two government hospitals in Jordan’s central region: Al-Basheer Hospital and Zarqa Governmental Hospital. The data were collected from individuals who regularly visited these hospitals for follow-up care or from their caregivers. The inclusion criteria were being Jordanian, aged 18 or older, able to communicate, and willing to participate. The exclusion criteria were being under 18, pregnant, or lactating.

### Ethical approvals and consent to participate

The study protocol was reviewed and approved by the Institutional Review Board of The Hashemite University and the Jordanian Ministry of Health (MOH/REC/2023/375). Before enrolling in the study, we obtained written informed consent from each willing and eligible subject.

### Sample size

The sample size was calculated using the Raosoft online calculator based on the 2020 Department of Statistics data. According to the data, adults comprise 52.2% (5,621,970) of Jordan’s population, which is 10,806,000. With a 5% margin of error, a 95% confidence interval, a 50% response distribution, and an additional 10% for contingencies, the minimum required sample size was 424 participants. However, 751 participants were recruited, with 95 males excluded as they did not represent a fair sample, and 656 females were included.

### Data collection

The data was collected using a questionnaire written in English, which was then translated into regional Arabic and back into English by a language expert to ensure accuracy. Additionally, pre-testing surveys were conducted using appropriate methodologies (reliability tests were performed to evaluate alpha values with various questionnaires, engaging 30 participants not part of the study. Alpha values for HLI and AEBQ were 0.81 and 0.87, respectively), and specialists were employed for each task to ensure the quality of the data. After the data-gathering process was complete, daily meetings were scheduled with data collectors to verify the data’s completeness, correctness, and consistency. A qualified nutritionist conducted participant interviews, gathered necessary data, and verified the accuracy of the collected anthropometric data under the supervision of the principal investigators.

### Sociodemographic data

Through the interviews, demographic data, including sex, marital status, employment status, educational level, family members, lifestyle data (such as sleep hours, smoking status, and physical activity), and public health and medication information were gathered.

### Anthropometric measurements

According to the standard procedure, body height was measured using Seca stadiometers (Seca, USA). Weight (kg), fat percentage, and muscle percentage were measured using bioelectrical impedance analysis (BIA) with the InBody H20N body fat scale (FSA, US) [18, 19]. Subjects were asked to wear light clothing and avoid wearing shoes during the measurements. The measurements were recorded to the nearest 0.1 cm and 0.1 kg. Body mass index (BMI) was also calculated in kilograms per square meter (kg/m²). Waist circumference (WC) and hip circumference (HC) were measured to the nearest 0.1 cm using RIEDER Body Measure tape, Lock Pin, and Push-Button Retract (Inct. Bonus Kit, REIDHK, China). The waist-to-hip ratio (WHR) was also calculated.

### Eating habits and dietary intake (lifeline diet score (LLDS))

The dietary intake was assessed by asking about the frequency of consumption of 12 food groups during the last seven days. The food groups were determined as follows: (1) whole grains, grains, rice, and pasta (2) potatoes; (3) milk and dairy products, (4) vegetables and leafy vegetables, (5) fruits, (6) legumes and nuts, (7) chicken and meats, (8) fish and tuna, (9) eggs, (10) desserts, honey, and snacks, (11) oils and fats, (12) spices and condiments [20]. The spices and condiments group received zero scores, while the other groups were categorized as having a negative, positive, neutral, or unknown impact on health. Positive groups included vegetables, fruits, whole-grain products, legumes, nuts, fish, and tuna. Negative food groups included red and processed meats, desserts, honey, snacks, oils, and fats. There were also unknown categories, such as potatoes, and a neutral group, which included eggs. The LLDS (Lifestyle-Dietary Score) was created using these positive and negative food groups. The frequency of intake was rated on a scale of 1 to 5, with 1 representing the lowest intake and 5 representing the highest intake [21]. The total score, known as the LLDS score, is the sum of the scores of the nine components. The LLDS score ranges from 9 to 45 and is categorized into quintiles as follows: ≤9, 10–18, 19–27, 28–36, and 37–45 [22]. The present sample of LLDS ranges from 18 to 34 (mean 24.9 ± 2.7).

### Healthy lifestyle index (HLI)

Please make a note of the following information. The Healthy Lifestyle Index (HLI) used in this analysis consists of five modifiable lifestyle factors: physical activity level, body fatness, smoking status, alcohol consumption, and diet. Each factor is assigned a score ranging from 0 to 4, with higher scores indicating a healthier lifestyle. Participants reported their physical activity level on a 10-point scale ranging from inactive to very active. The physical activity level was then scored by quintile, with the highest quintile receiving a score of 4 and the lowest quintile receiving a score of 0 [23]. Body fatness was assessed using Body Mass Index (BMI), with the following scores: BMI < 23 = 4, 23 to < 25 = 3, 25 to < 27 = 2, 27 to < 30 = 1, and BMI ≥ 30 = 0. Smoking status was scored based on intensity and time since cessation, with the following scores: never smoker = 4, former smoker > ten years since cessation = 3, former smoker ≤ 10 years since cessation = 2, smoker < 15 cigarettes/day = 1, and current smoker ≥ 15 cigarettes/day = 0. It’s worth noting that in this study, alcohol consumption was not considered a lifestyle factor due to the cultural context. Jordan, where the study was conducted, is a Muslim country, and alcohol consumption is not a common lifestyle choice among Jordanians. Every participant received a score of 4 for their alcohol consumption, which is the maximum score for those who drink less than 6 g of alcohol per day. Participant scores for each lifestyle factor were summed up to obtain the HLI score, which varied from 0 to 20. Another methodology used WHR (HLI_WHR_) rather than BMI (HLI _BMI_); as WHR is a stronger link to central obesity [23].

### Adult eating behavior questionnaire (AEBQ)

The AEBQ is a 35-item questionnaire that evaluates the “Food Approach” and “Food Avoidance” subscales. Food approach subscales involve food responsiveness (4 questions), hunger (5 questions), emotional overeating (5 questions), and enjoyment of food (3 questions) [24]. Food avoidance subscales include satiety responsiveness (4 questions), food fussiness (5 questions), emotional undereating (5 questions), and slowness in eating (4 questions) [24]. Participants were assessed to rate their responses on a 5-point Likert scale ranging from 1 (strongly disagree) to 5 (strongly agree). Subscale mean scores were then calculated [25, 26].

### Blood pressure measurements

The hospital’s protocols required a digital BP monitor (A&D Medical, UA-7675 digital BP monitor, Japan) to assess blood pressure. Before the measurement, participants were given a 30-minute relaxation period during which they were seated with uncrossed legs and refrained from consuming caffeine or smoking. They abstained from drinking coffee and smoking for 120 min before the measurement [27]. The participant’s left upper arm was positioned at the same level as the heart, often the preferred arm for taking blood pressure readings. Following this, three seated blood pressure readings were taken, with each reading spaced one to five minutes apart to prevent hyperemia in the upper arm [28, 29].

### Biochemical measurements

The Hakim online medical system collected fasting blood glucose (FBG) levels. Serum lipid profile data, in mmol/L (including HDL and TG), were collected from Hakim’s online medical system.

### Metabolic syndrome (MetS) components

National Cholesterol Education Program Adult Treatment Panel III (NCEP-ATPIII) determined the diagnostic criteria of MetS: If three or more of the following five abnormal values of the MetS components are diagnosed for each participant: enlarged WC; ≥ 88 cm, FBG ≥ 6.1 mmol/L or using of anti-diabetic medication, elevated BP; SBP ≥ 130 mmHg or DBP ≥ 85 mmHg or on BP- medication, TG ≥ 1.7 mmol/L or using medication for raised TG, and HDL.

< 1.29 mmol/L or taking medication for reduced HDL [30].

### Statistical analysis

Analyses were done using SPSS software (IBM SPSS Statistics for Windows, Version 23.0. Armonk, NY: IBM Corp). Descriptive statistical tests were used to calculate frequencies and percentages for general characteristics. The chi-square test was used to assess differences in proportions. Spearman’s rho test assessed MetS, HLIBMI, HLIWHR, and AEBQ coloration. The *p*-value of less than 0.05 reflected statistical significance.

## Results

A total of 656 female participants were included in this study. As shown in Table 1, the participants had a mean weight of 78.36 ± 17.37 kg, a height of 159.95 ± 6.00 cm, a BMI of 30.71 ± 6.56 kg/m^2^, a WC of 96.77 ± 14.72 cm, an HC of 112.00 ± 15.16 cm, a % Body fat of 41.65 ± 7.94, a % Body muscle mass of 24.46 ± 4.22, an SBP of 118.82 ± 19.34 mmHg, a DBP of 80.77 ± 11.99 mmHg, blood glucose of 7.09 ± 3.93 mmol/L, HDL of 1.29 ± 0.44 mmol/L, TG of 1.78 ± 1.11 mmol/L. 23.5% of the participants were aged 19–35, 42.2% were 36–50, and 34.3% were 51–65. Almost three-quarters of the study sample held primary, secondary, or high school education levels, and 75.8% were married. In addition, half of the participants were obese, and 29.9% of them were overweight. The prevalence of smoking was 31.3%, and 38.7% were passive smokers. Of the 656 participants, 62.6% were physically inactive. The participants with MetS were 234 (35.7%) vs. 422 without MetS.

**Table 1:** Description of sample general characteristics (*n* = 656)

Variables	Mean ± SD
Weight (kg)	78.36 ± 17.37
Height (cm)	159.95 ± 6.00
Body mass index (kg/m^2^)	30.71 ± 6.56
Waist circumference (cm)	96.77 ± 14.72
Hip circumference (cm)	112.00 ± 15.16
% Body fat	41.65 ± 7.94
% Body muscle mass	24.46 ± 4.22
Systolic BP (mmHg)	118.82 ± 19.34
Diastolic BP (mmHg)	80.77 ± 11.99
Blood glucose (mmol/L)	7.09 ± 3.93
HDL (mmol/L)	1.29 ± 0.44
TG (mmol/L)	1.78 ± 1.11
	**n (%)**
**Age group**	
19–35 years	154 (23.5)
36–50 years	277 (42.2)
51–65 years	225 (34.3)
**Educational level**	
Primary and secondary school	191 (29.1)
High school	292 (44.5)
Student (college/university)	94 (14.3)
B.Sc. degree and Higher education (master’s/Ph. D.)	79 (12.1)
**Marital status**	
Married	497 (75.8)
Single	97 (14.8)
Divorced	25 (3.8)
Widow	37 (5.6)
**Smoking status**	
Nonsmoker	161 (24.5)
Smoker (Cigarette and/or hookah)	205 (31.3)
Passive smoker	254 (38.7)
Ex-smoker	36 (5.5)
**Body Mass Index categories**	
Underweight	11 (1.8)
Normal weight	106 (17.2)
Overweight	184 (29.9)
Obese	315 (51.1)
**Physical activity**	
No	408 (62.2)
Yes	248 (37.8)

The association between MetS and HLI_BMI_, HLI_WHR_, and AEBQ is presented as a mean and standard deviation, as shown in Table 2. The participants with MetS had a significantly higher percentage of body fat (44.15 ± 6.37) and, interestingly, a higher rate of body muscle mass (25.7 ± 4.56) compared to normal participants (40.28 ± 8.38, *p* < 0.001 and 23.8 ± 3.87, *p* = 0.002, respectively). The mean HLI_BMI_ or HLI_WHR_ for participants with MetS (10.7 ± 1.51 and 11.6 ± 2.03, respectively) was significantly lower than that for normal participants (11.5 ± 2.37, *p* = 0.003, and 12.4 ± 2.37, *p* = 0.004, respectively). Regarding AEBQ, there was no significant difference between participants with MetS and those who were healthy.

**Table 2:** The association between MetS and HLI_BMI_, HLI_WHR_, and AEBQ (*n* = 656)

Variables	MetS (Mean ± SD)		*p*-value*
	Absence (*n* = 422)	Presence (*n* = 234)	
% Body fat	40.28 ± 8.38	44.15 ± 6.37	< 0.001
% Body muscle mass	23.8 ± 3.87	25.7 ± 4.56	0.002
HLI_BMI_	11.5 ± 2.37	10.7 ± 1.51	0.003
HLI_WHR_	12.4 ± 2.37	11.6 ± 2.03	0.004
AEBQ	106.2 ± 10.44	106.0 ± 10.10	0.830

MetS: metabolic syndrome; HLI: Healthy Lifestyle Index; BMI: Body mass index; WHR: waist to hip ratio; and AEBQ: Adult Eating Behavior questionnaires

* Value < 0.05 considered significant

Using Spearman’s rho test (Table 3), a significant weak correlation was found between MetS and the percentage of body fat (*r* = 0.233, *p* < 0.001) and the percentage of body muscle mass (*r* = 0.210, *p* < 0.001). Additionally, a significant negative, very weak correlation was found between MetS and HLIBMI (*r* = -0.085, *p* = 0.036). Studying the correlation between MetS component numbers and the same variables revealed a moderately significant correlation between the number of MetS components and the percentage of body fat (*r* = 0.355, *p* < 0.001) and the percentage of body muscle mass (*r* = 0.325, *p* < 0.001). Additionally, a weak inverse correlation was observed between the number of MetS components and HLI_BMI_ (*r* = -0.156, *p* < 0.001) and HLI_WHR_ (*r* = -0.103, *p* = 0.008). Moreover, there was a weak inverse correlation between HLI_BMI_ and the percentage of body fat (*r* = -0.154, *p* < 0.001) and the percentage of body muscle mass (*r* = -0.200, *p* < 0.001). There was no correlation between AEBQ and any of the variables.

**Table 3:** The correlation between MetS, MetS components, HLI_BMI_, HLI_WHR_, and AEBQ

Variables		MetS	MetS component	% Body fat	% Body muscle mass	HLI_BMI_	HLI_WHR_	AEBQ
MetS	r		0.851**	0.227^**^	0.215^**^	-0.085^*^	-0.059	− 0.018
	*p*-value		< 0.001	< 0.001	< 0.001	0.036	0.132	0.651
MetS component	r	0.851**		0.355^**^	0.325^**^	-0.156^**^	-0.103^**^	0.037
	*p*-value	< 0.001		< 0.001	< 0.001	< 0.001	0.008	0.350
% Body fat	r	**0.233** ^**^	**0.355** ^**^		0.272^**^	**-0.154** ^**^	-0.021	-0.025
	*p*-value	**< 0.001**	**< 0.001**		< 0.001	**< 0.001**	0.600	0.518
% Body muscle mass	r	**0.210** ^**^	**0.325** ^**^	0.272^**^		**-0.200** ^**^	-0.005	0.035
	*p*-value	**< 0.001**	**< 0.001**	< 0.001		**< 0.001**	0.905	0.371
HLI_BMI_	r	**-0.085** ^*^	**-0.156** ^**^	-0.154^**^	-0.200^**^		0.658^**^	-0.071
	*p*-value	**0.036**	**< 0.001**	< 0.001	< 0.001		< 0.001	0.078
HLI_WHR_	r	-0.059	**-0.103** ^**^	-0.021	-0.005	0.658^**^		-0.073
	*p*-value	0.132	**0.008**	0.600	0.905	< 0.001		0.063
AEBQ	r	-0.018	0.037	-0.025	0.035	-0.071	-0.073	
	*p*-value	0.651	0.350	0.518	0.371	0.078	0.063	

MetS: Metabolic syndrome; HLI: Healthy lifestyle index; BMI: Body mass index; WHR: waist to hip ratio; AEBQ: Adult eating behavior questionnaires

**. Correlation is significant at the 0.01 level (2-tailed)

*. Correlation is significant at the 0.05 level (2-tailed)

The association based on the number of abnormal MetS components with HLI_BMI_, HLI_WHR_, and AEBQ is illustrated in Table 4. As MetS components increased, the mean percentage % of body fat and % of body muscle mass increased. In more detail, participants with five- components had significantly the highest % of body fat (46.07 ± 4.80, *p <* 0.001), which insignificantly differed from participants with two, three, and four components’ participants, while significantly differed from participants with one abnormal component (40.46 ± 7.64, *p <* 0.001) and zero component, which had significantly the lowest mean (34.24 ± 8.00, *p <* 0.001). Furthermore, participants with five abnormal components had the highest % of body muscle mass (27.85 ± 4.54, *p <* 0.001), which significantly differed from participants with four, three, two, one, and zero abnormal components that had significantly the lowest mean (21.76 ± 3.43, *p =* 0.002; respectively). Regarding HLI_BMI_ and HLI_WHR_, the mean decreased significantly with the increasing number of MetS components. In addition, the participants with zero abnormal components had significantly higher HLI_BMI_ (12.97 ± 2.48, *p =* 0.003) and HLI_WHR_ (13.73 ± 2.39, *p =* 0.004) in comparison to participants with one (11.48 ± 2.25 and 12.22 ± 2.27, respectively), two (10.72 ± 2.02 and 11.89 ± 2.20, respectively), three (10.54 ± 1.50 and 11.51 ± 1.95, respectively) four (10.95 ± 1.95 and 11.72 ± 2.34, respectively) or five abnormal components (10.40 ± 0.52 and 11.09 ± 0.94, respectively). There were no significant differences in AEBQ scores between the participants with different numbers of MetS components.

**Table 4:** **T**he association between the MetS component and HLI_BMI_, HLI_WHR_, and AEBQ (*n* = 656)

Variables	Mean ± SD						*p*-value*
	Zero-component (*n* = 92)	One-component (*n* = 154)	Two-component (*n* = 176)	Three-component (*n* = 157)	Four-components (*n* = 66)	Five-components (*n* = 11)	
% Body fat	34.24 ± 8.00	40.46 ± 7.64	43.29 ± 7.50	44.11 ± 6.50	43.96 ± 6.31	46.07 ± 4.80	< 0.001
% Body muscle mass	21.76 ± 3.43	23.63 ± 3.83	25.02 ± 3.65	25.65 ± 4.84	25.33 ± 3.78	27.85 ± 4.54	0.002
HLI_BMI_	12.97 ± 2.48	11.48 ± 2.25	10.72 ± 2.02	10.54 ± 1.50	10.95 ± 1.95	10.40 ± 0.52	0.003
HLI_WHR_	13.73 ± 2.39	12.22 ± 2.27	11.89 ± 2.20	11.51 ± 1.95	11.72 ± 2.34	11.09 ± 0.94	0.004
AEBQ	104.71 ± 8.82	105.49 ± 10.98	107.67 ± 10.60	105.95 ± 8.71	105.33 ± 12.46	111.73 ± 11.97	0.076

MetS: Metabolic syndrome; HLI: Healthy lifestyle index; BMI: Body mass index; WHR: waist to hip ratio; AEBQ: Adult eating behavior questionnaires

* Value < 0.05 considered significant

## Discussion

The incidence of MetS, which is associated with an increased risk of non-communicable diseases, is steadily rising in tandem with the rise in obesity. The relative influence of modifiable risk factors for MetS. The present study investigated the association between HLI_BMI_, HLI_WHR_, AEBQ, and MetS components among female Jordanian adults.

The current findings revealed that the participant with MetS had a significantly higher % of body fat and a higher % of body muscle mass, which is interesting, compared to participants without MetS. Also, as the number of MetS components increased, the mean percentage % of body fat and % of body muscle mass increased. Studying the correlation of MetS components revealed that there was a moderately significant correlation between the MetS component number and % of body fat (*r* = 0.355, *p <* 0.001) and % of body muscle mass (*r* = 0.325, *p <* 0.001). This was not surprising; it has been found that higher FMI levels seem to be positively linked with MetS presence, regardless of BMI and body fat [9]. In concordance with our findings, it has been approved that the number of MetS components was directly correlated to weight, body mass index, fat mass, lean mass, and visceral fat area, and the severity of the MetS is connected to body composition, inflammation, and visceral adiposity in women [8]. According to Grundy, the development of MetS may be significantly influenced by extra adipose tissue [3].

Additionally, body fat% was significantly higher in participants with MetS and T2D versus those without MetS and T2D (*p* < 0.05) [31]. After adjusting for smoking status, age, exercise status, and alcohol consumption, the men with higher than normal body fat percentage had an odds ratio of 3.61 (95% confidence interval, 2.10–6.22; *p* < 0.001) for developing MetS, whereas women had an odds ratio of 1.95 (95% CI, 1.30–2.92; *p* < 0.001) [32]. Both fat-free mass index (FFMI) and FMI were significantly correlated to higher odds of MetS (OR 3.97, for FMI; OR 2.67, for FFMI) [33].

Moreover, Kown and colleagues observed a significant positive association between the area of visceral adipose tissue and a higher incidence of MetS. Subcutaneous adipose tissue may protect against the incidence of specific MetS components, even though the visceral adipose tissue area was longitudinally linked with the incidence of each MetS component [34]. Also, body fat and FMI were positively correlated to MetS components (*p* < 0.05). Body fat% and FMI thresholds of 25.55% and 6.97 kg/m^2^ in males and 38.95% and 11.86 kg/m^2^ in females indicated elevated MetS risk [35].

In the Shukohifar et al. (2022) study, they concluded that both body fat% and BMI ought to be well-thought-out in screening phases; in detail, body fat% could be regarded as a better predictor of hyperglycemia in the male group, while it was a better predictor of hypertension, hypertriglyceridemia, and hypo HDL than BMI in the women group [36].

Results were conflicting regarding muscle mass and MetS occurrence; Kim and colleagues found a protective association between muscle mass and MetS in Korean adults. After adjustment for potential confounders, high muscle/low fat was correlated with lower insulin resistance (*P* < *0.001*) than low muscle/low fat. Low muscle/high fat and high muscle/high fat were significantly correlated with the prevalence of MetS [10], which agreed with the present findings. On the other hand, Song and colleagues conclude a significant graded inverse association between muscle mass and MetS. In the components of MetS, muscle mass was significantly inversely associated with high WC and elevated BP (*p* < 0.05), affecting physical performance, which was not linked with components of MetS [37]. Our findings show that low muscle mass increased the risk of MetS significantly after adjusting for possible covariates (OR, 5.28; 95% CI, 2.76–10.13) in adolescents. Similarly, the ORs for the MetS components were significantly lower in subjects with high muscle mass than those with low muscle mass [38]. From another point of view, Lee and colleagues accurately mapped the amount of fat and the skeletal muscle mass using computed tomography (CT). They concluded that muscle quality and quantity were correlated to the prevalence of MetS in both sexes. Though “the total abdominal muscle area (TAMA) showed a stronger association with MetS in males than in females compared to low-attenuation abdominal muscle area (LAMA), normal-attenuation abdominal muscle area (NAMA), and extramyocellular lipid area (EMCLA)” [39].

The present findings indicated that the mean HLI_BMI_ or HLI_WHR_ for subjects with MetS was significantly lower than for participants without MetS. Also, a weak inverse correlation was reported between the number of MetS components with HLI_BMI_ (*r*=-0.156, *p <* 0.001) and HLI_WHR_ (*r*=-0.103, *p =* 0.008). Regarding HLI_BMI_ and HLI_WHR_, the mean decreased significantly with the increasing number of MetS components. In agreement with this, Niu and colleagues (2023) found that for those with MetS and traits similar to it, adhering to an apparently healthy lifestyle can lower the risk of death causes; the more favorable the effect, the higher the score [40]. Also, Mirmiran and colleagues (2022) investigated the connection between HLS and the risk of MetS in Tehranian adults; they concluded a negative correlation between the higher score of HLS-AHEI-2010 with the risk of MetS (*p* for trend < 0.05), also they found that the risk of MetS decreased across tertiles of HLS-AHEI-2010 [15]. Moreover, as expected to approve our findings, it has been found that higher observance of a healthy lifestyle was linked to a lower risk of developing MetS in Spain [41]. In Iranian women, those in the highest Health Lifestyle Score (HLS) quartile had lower WC, BMI, and weight. They also had lower FBG and TG than those in the lowest quartile of HLS. Additionally, women in the second quartile of HLS had 24% higher odds of having MetS than those in the first quartile [42].

As mentioned in the materials and methodology, the HLI components were physical activity, smoking status, WC, body weight, dietary intake, and alcohol consumption (presented by LLDS). Nevertheless, because lifestyle factors are often highly interrelated, looking at lifestyle as a whole rather than a unit variable is more beneficial. Our results aligned with several earlier research highlighting the advantageous effects of adopting various healthy lifestyle practices in the early prevention of MetS. These studies showed that the risk of MetS reduced with an increase in healthy lifestyle factors, indicating that a combination of factors, rather than a single factor, is more likely to be correlated with lower MetS risks [11, 12, 43]. In a study conducted in northern Taiwan, Chang and colleagues (2016) discovered that obese individuals who exercised regularly and consumed sufficient amounts of fruit were less likely to have MetS. Additionally, overweight nonsmokers who consumed adequate amounts of vegetables were also less likely to have MetS [12]. Furthermore, among 1408 Korean men aged 20–75, 1/3 of the 30–49 age group had ≥ 3 combined deprived lifestyle factors; Lee and colleagues concluded that the ORs for MetS were increased as components of poor lifestyle factors increased from 3.57 (95% CI 1.16–11.02) for subjects with single poor factor, 3.62 (95% CI 1.18–11.08) for subjects with two poor lifestyle factors, to 6.31 (95% CI 2.08–19.26) in subjects with three or more poor lifestyle factors, respectively; compared with those with no poor lifestyle factors. It was obvious that the current smoking, high-risk alcohol use, and high physical inactivity were present, and the ORs for MetS, hypertriglyceridemia, and hyperglycemia were increased [11]. Also, Buscemi et al. (2014) revealed that MetS was associated with age and physical activity level and suggested that an insufficient physical activity level is associated with MetS [44]. Another study by VanWormer et al. found that a higher risk of MetS is related to a decrease in healthy lifestyle characteristics over two years, primarily increased weight, alcohol drinking, and lower vegetable/fruit intake [13]. Adherence to good lifestyle factors, such as a nutritious diet, abstinence from alcohol, physical exercise, and smoking, has also been shown in many cohort and cross-sectional studies to reduce the risk of MetS in males over 55 [13, 14]. In contrast, among African Americans in the USA, it has been found that while age and marital status were substantial predictors of MetS, lifestyle factors such as WC, WHR, BMI, smoking, and physical activity did not significantly correlate with MetS [45].

The present study considers the HLI_WHR_, as it is commonly known that central obesity, which gonadal hormones may influence, is a hallmark of MetS. It has been approved that WHR was the highest predictor for MetS components [46]. Also, the most common aspect of MetS is abdominal obesity [47]. Moreover, numerous cross-sectional studies assert that visceral adipose tissue enhances the risk of MetS in various racial and ethnic groups [48, 49].

Finally, higher HLI scores are linked to lower levels of body fat (percent body fat, body fat mass), waist circumference, waist-to-hip ratio, and BMI, while increasing fat-free mass (lean tissue) in a dose-dependent manner [50, 51]. Also, higher HLI scores are associated with substantial reductions in the risk of cardiovascular disease (up to 52% lower risk in the highest vs. lowest HLI quintile), stroke, coronary heart disease, and related events—even among individuals with normal BMI [52]. Thus, healthy lifestyle scores have the potential to serve as instruments for health promotion, assisting individuals in making health-conscious decisions about their behavior. More studies in various contexts would be beneficial in examining the possible impacts of HLI more thoroughly on healthy habits and health outcomes. The magnitude of differences in HLI scores is clinically significant, leading to healthier body composition and marked reductions in the risk of obesity, cardiovascular disease, cancer, and multimorbidity. Adopting a healthier lifestyle, as reflected by higher HLI scores, can have a profound and measurable impact on long-term health outcomes.

The study’s main strengths were rich information resources and numerous recorded and calculated measures for evaluation. Moreover, all data were collected by trained interviewers with reliable and valid tools. In some research, healthy lifestyle factors were collected in parallel with the AEBQ, which covers the stress factor associated with MetS. Nonetheless, there are some limitations, such as the nature of cross-sectional studies, which do not lead to a causality relationship; the male sample was low and not representative, so they were excluded, recruiting from otclinics in the hospital settings, and the potential for selection bias. Moreover, a limitation could be addressing using BIA InBody; it consistently shows systematic bias: they tend to underestimate BF% and fat mass, and overestimate FFM when compared to dual-energy X-ray absorptiometry (DXA), which is considered a gold standard [53, 54]. Therefore, our findings may not apply to the general population, and further research is necessary, particularly for males.

## Conclusion

The current findings suggest that as the number of MetS components increases, the percentage of body fat and body muscle mass also increases. This indicates a potential link between MetS and body composition. However, the scores for HL_IBMI_ and HLI_WHR_, indicators of a healthy lifestyle, decrease significantly with the increasing number of MetS components. This underscores the importance of maintaining a healthy lifestyle to prevent or manage MetS. Interestingly, the AEB, which measures eating behavior, was not affected by the number of MetS components. This suggests that dietary habits may not significantly affect MetS development (see the graphical abstract).

## Data Availability

No datasets were generated or analysed during the current study.
